# Fully human antagonistic antibodies targeting FPR2 through dual extracellular-loop engagement for gastric cancer therapy

**DOI:** 10.1093/abt/tbag035

**Published:** 2026-07-03

**Authors:** Min Su Kim, Tae Hyun Kang

**Affiliations:** Department of Biopharmaceutical Chemistry, Kookmin University, Seoul, 02707, Republic of Korea; Antibody Research Institute, Kookmin University, Seoul, 02707, Republic of Korea; Department of Biopharmaceutical Chemistry, Kookmin University, Seoul, 02707, Republic of Korea; Antibody Research Institute, Kookmin University, Seoul, 02707, Republic of Korea; Biopharmaceutical Chemistry Major School of Applied Chemistry, Kookmin University, Seoul, 02707, Republic of Korea; Department of Pharmaceutical Engineering, Kookmin University, Seoul, 02707, Republic of Korea

**Keywords:** N-formyl peptide receptor 2, therapeutic antibody, GPCR antibody discovery, gastric cancer, antibody engineering

## Abstract

**Background:**

Gastric cancer (GC) remains a major cause of cancer mortality worldwide, particularly in metastatic stages. N-formyl peptide receptor 2 (FPR2), a class A G protein-coupled receptor activated by the *Helicobacter pylori*–derived peptide Hp(2–20), promotes GC progression by stimulating oncogenic signaling pathways. However, no clinically applicable agents selectively targeting FPR2 have been developed.

**Methods:**

We generated fully human antagonistic antibodies targeting FPR2 using a two-step discovery strategy. A CDR-H3–focused synthetic single-chain variable fragment library was screened against an extracellular loop 3 (ECL3) peptide, followed by light-chain engineering to confer dual engagement of extracellular loop 2 (ECL2).

**Results:**

The engineered antibody exhibited dose-dependent binding to both ECL2 and ECL3 peptides, selectively recognized FPR2 over other FPR family members, and showed nanomolar binding to FPR2-expressing cancer cells. The antibody inhibited Hp(2–20)–induced calcium mobilization and suppressed migration and invasion of Adenocarcinoma Gastric (AGS) gastric cancer cells. Structural modeling indicated a lid-like binding mode occluding the orthosteric pocket.

**Conclusions:**

We developed a fully human antagonistic antibody that selectively binds and inhibits FPR2 through dual extracellular-loop engagement. The antibody potentially suppresses FPR2-driven signaling and invasive behaviors in gastric cancer cells, supporting its development for therapeutic application. Further assessment of its efficacy *in vivo* is warranted.

## Graphical Abstract

**Graphical Abstract:**
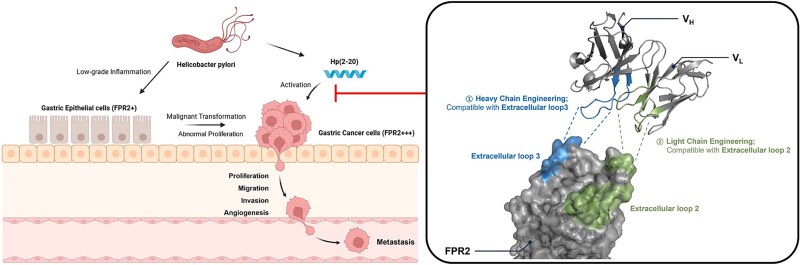

## Teaser

Statement of Significance This study describes the development of a fully human monoclonal antibody engineered to antagonize the G protein-coupled receptor N-formyl peptide receptor 2 (FPR2) through recognition of two ECLs. The antibody suppresses FPR2-mediated calcium signaling as well as migration and invasion of gastric cancer cells, supporting its potential as a therapeutic candidate targeting FPR2-driven tumor progression.

## Introduction

Gastric cancer (GC) remains one of the leading causes of cancer-related mortality [1]. Although early detection and multimodal therapy have improved outcomes, the prognosis for patients with advanced or metastatic GC remains extremely poor, with 5-year survival rates below 10% [2]. Thus, novel therapeutic strategies that inhibit metastatic dissemination are urgently needed.

N-formyl peptide receptor 2 (FPR2), a class A G protein-coupled receptor (GPCR), is traditionally known for its immunomodulatory roles [3, 4]. However, accumulating evidence demonstrates that aberrant overexpression of FPR2 in GC confers a pro-tumorigenic phenotype. Activation of FPR2 by Hp(2–20) [5, 6], a *Helicobacter pylori*–derived peptide, induces Mitogen-Activated Protein Kinase/Extracellular Signal-Regulated Kinase (MAPK/ERK) signaling, promotes epithelial–mesenchymal transition, and invasion [7–11]. Inhibition of FPR2 suppresses tumor progression in preclinical models, highlighting FPR2 as a compelling therapeutic target [7].

Despite its pathological relevance, clinically approved therapeutics that selectively target FPR2 remain unavailable, largely due to structural constraints inherent to GPCRs, including high sequence homology to other FPR family members, FPR1 and FPR3, necessitating exceptional target specificity to minimize off-target activity [3, 12]. In this regard, antibody-based modalities offer high affinity and specificity, favorable pharmacokinetics, and the potential to leverage Fc-mediated effector functions when desired [13, 14]. However, as a GPCR, FPR2 presents intrinsic barriers to antibody development, including seven transmembrane helices, conformational plasticity, and limited extracellular surface area [15]. These characteristics make it difficult to maintain the receptor in a stable, native conformation during solubilization, thereby complicating antigen presentation and constraining conventional antibody discovery approaches [16–18].

In GPCR antibody discovery, peptide-based antigens mimicking extracellular loops (ECLs) have been widely used as a pragmatic alternative to full-length receptor antigens [19–22]. This approach enables direct targeting of extracellular domains without requiring preparation of intact receptors. However, reliance on a single linear peptide may inherently limit epitope diversity and bias discovery away from conformational epitopes that are critical for receptor function, potentially yielding antibodies that recognize peptide fragments yet exhibit limited binding or functional activity against the native receptor in its membrane context [23, 24].

Building upon these considerations, we employed two distinct ECL-mimicking peptides as antigens to broaden epitope coverage beyond a single linear loop, thereby facilitating the discovery of fully human antagonistic antibodies against FPR2 with the goal of suppressing FPR2-driven GC progression. To accomplish this, we rationally designed heavy-chain complementarity-determining region 3 (CDR) loops to recognize the ECL3 peptide and subsequently diversified light-chain CDR loops to engage the ECL2 peptide through phage display–based selection. Through this strategy, we isolated an anti-FPR2 antibody exhibiting dual recognition of FPR2 ECL2 and ECL3 with low-nanomolar affinity and selective binding to cell-surface FPR2, with minimal reactivity toward FPR1 and FPR3. The engineered antibody bound both FPR2-overexpressing CHO-K1 cells and endogenous FPR2-positive GC cells with nanomolar EC_50_ values. Functionally, the antibody inhibited Hp(2–20)–induced FPR2 activation and downstream calcium signaling in a calcium reporter assay and significantly suppressed migration and invasion of human GC cells. Finally, *in silico* docking simulations suggested that the engineered antibody should engage the ECLs of FPR2 in a lid-like manner over the ligand-binding pocket, providing a structural rationale for its antagonistic activity.

## Materials and methods

### Cell lines

CHO-K1 and AGS cells were obtained from American Type Culture Collection (ATCC, Manassas, USA) and cultured in Ham’s F-12 Nutrient Mix supplemented with GlutaMAX™ and Kaighn’s Modification of Ham’s F-12 (F-12K), respectively (Gibco, Thermo Fisher Scientific, USA). Media were supplemented with 10% fetal bovine serum (FBS; Gibco, Thermo Fisher Scientific, USA) and 1% penicillin–streptomycin (P/S; Gibco, Thermo Fisher Scientific, USA). Cells were incubated at 37°C with 5% CO₂. For routine passage, cells at ~80% confluence were detached using 0.25% trypsin–EDTA (Gibco, Thermo Fisher Scientific, USA) or phosphate-buffered saline (PBS) containing 0.5 mM EDTA.

### Antibody library construction

A synthetic human single-chain variable fragment (scFv) library was constructed through CDR-targeted mutagenesis Polymerase Chain Reaction (PCR), overlap-extension assembly, subcloning into a proofreading vector, transfer to a phagemid backbone, and subsequent phage rescue.

PCR amplification was performed using human germline VH3-23 and Vκ1-39 scaffolds with primer sets designed for CDR-specific diversification and Variable Light chain (V_L_)-diversified scFv library construction. Reactions were carried out under the standard PCR conditions. Purified V_H_ and V_L_ fragments were assembled by overlap-extension PCR to generate full-length scFv inserts flanked by SfiI restriction sites, followed by SfiI digestion and PCR purification. SfiI-digested scFv fragments were ligated into SfiI-linearized pBLA proofreading vectors using T4 DNA ligase [25]. Ligation mixtures were desalted and electroporated into freshly prepared *Escherichia coli* Jude-1 electrocompetent cells. After recovery in Super Optimal broth with Catabolite repression (SOC) medium, cells were plated on LB agar supplemented with isopropyl β-D-1-thiogalactopyranoside (IPTG), carbenicillin, and chloramphenicol. Colonies were harvested after overnight incubation at 37°C. In-frame scFv inserts were verified by plasmid isolation and SfiI digestion, excised, and ligated into SfiI-linearized pComb3X phagemid vectors [26]. The ligation products were electroporated into *E. coli* ER2738 cells, recovered in SOC medium, and plated on carbenicillin-selective agar plates. Colonies were scraped, pooled, and stored as glycerol stocks (final 15%, v/v) at −80°C to establish the phage-display scFv library.

For phage rescue, the ER2738 library stock was inoculated into 400 ml Super Broth (SB) supplemented with appropriate antibiotics and 2% glucose and cultured at 37°C to an OD_600_ of ~0.5. Cells were pelleted, resuspended in fresh SB medium without glucose, and infected with M13KO7 helper phage at a multiplicity of infection (MOI) of 10. Infection proceeded at 37°C for 1 h with gentle agitation, followed by addition of kanamycin and overnight incubation at 30°C for phage amplification. The bacterial cells were removed by centrifugation, and phage particles in the supernatant were precipitated with Polyethylene Glycol (PEG)–NaCl solution on ice. Phages were pelleted by centrifugation, resuspended in PBS, clarified by centrifugation, and filtered through a 0.22 μm membrane. Phage titers were determined by serial dilution, and purified phages were stored in 15% (v/v) glycerol at −80°C until use.

### Biopanning

Phage display biopanning was performed to isolate scFv clones specific to FPR2 ECL peptides. Synthetic ECL2 (corresponding to residues 165–185 of human FPR2) and ECL3 (corresponding to residues 268–276 of human FPR2) peptides (GenScript, USA; CSbio, USA) were conjugated to CNBr-activated Sepharose 4B resin (Cytiva, Sweden) (Supplementary Table S6). For antigen coupling, CNBr-activated resin was washed with 1 mM HCl and equilibrated in coupling buffer (0.1 M NaHCO₃, 0.5 M NaCl, pH 8.3). Synthetic ECL peptide (50 μg) was incubated with 700 μl of resin suspension overnight at 4°C under gentle rotation. The coupled resin was washed with PBS, blocked with 0.1 M Tris–HCl (pH 8.0) for 2 h at room temperature, washed again, and stored in PBS at 4°C until use. Each panning round used peptide-coupled resin blocked with 3% bovine serum albumin (BSA) in PBS for 2 h at room temperature. Approximately 200 μl of phage library diluted in PBS containing 1% BSA was incubated with the resin overnight at 4°C. After washes of increasing stringency, bound phages were eluted with 100 mM glycine (pH 2.2) containing 1% BSA and neutralized with 1 M Tris–HCl (pH 8.0). Eluted phages were amplified in mid-log phase *E. coli* ER2738 (OD_600_ ≈ 0.6) and rescued using M13KO7 helper phage (MOI 5; New England Biolabs, UK). Cultures were incubated overnight at 30°C in SB medium containing carbenicillin and kanamycin. Phage particles were precipitated using 4% PEG 8000 (w/v) and 3% NaCl (w/v), resuspended in PBS containing 1% BSA, filtered (0.22 μm), and used for subsequent selection rounds or stored at −80°C in 15% glycerol.

### Expression and purification of antibodies

scFv constructs in pComb3X were transformed into *E. coli* TOP10F′ competent cells. Single colonies were grown overnight in SB supplemented with carbenicillin at 37°C with shaking (180 rpm) and subsequently inoculated into fresh SB medium. Cultures were grown at 37°C to an OD_600_ of 0.6–0.8, and scFv expression was induced with 1 mM IPTG followed by incubation at 30°C for 16 h. Cells were harvested and subjected to periplasmic extraction using TES buffer (20% sucrose, 50 mM Tris–HCl pH 8.0, 1 mM EDTA) supplemented with 1 mg/ml of lysozyme (Sigma-Aldrich, USA), followed by MgCl₂ addition to quench residual EDTA. The clarified periplasmic fraction was applied to Ni–NTA agarose resin (Thermo Scientific, USA) pre-equilibrated in Phosphate-Buffered Saline with Tween 20 (PBST) (PBS containing 0.05% Tween-20). After washing with PBST containing 20 mM imidazole, bound scFv was eluted with 200 mM imidazole in PBST. Full-length human Immunoglobulin G (IgG) antibodies were transiently expressed in Expi293F cells (Thermo Fisher Scientific, USA) by co-transfection of heavy- and light-chain expression vectors according to the manufacturer’s protocol. Cultures were maintained under standard shaking conditions, and supernatants were harvested 7 days post-transfection and clarified by centrifugation and 0.45 μm filtration. IgGs were purified by Protein A (GenScript, USA) affinity chromatography. Purified antibodies were analyzed using Sodium Dodecyl Sulfate - Polyacrylamide Gel Electrophoresis (SDS–PAGE), buffer-exchanged into PBS, and stored at −80°C.

### Enzyme-linked immunosorbent assay

Binding of purified scFv and IgG antibodies to synthetic human FPR2 ECL2 and ECL3 peptides was evaluated by enzyme-linked immunosorbent assay (ELISA). High-binding 96-well plates were coated overnight at 4°C with 200 ng/well of peptide in 0.05 M sodium carbonate buffer (pH 9.6). Plates were washed with PBS and blocked with 5% (w/v) skim milk in PBST (0.05% Tween-20) for 2 h at room temperature. Purified scFv or IgG samples were diluted in PBST and incubated for 2 h at room temperature. After washing with PBST, bound scFvs were detected using Horseradish Peroxidase (HRP)-conjugated anti-HA antibody (Thermo Fisher Scientific, USA), whereas bound IgGs were detected using HRP-conjugated goat anti-human kappa light-chain antibody (Thermo Fisher Scientific, USA). The reaction was developed using TMB substrate and stopped with 2 N H₂SO₄, and absorbance was measured at 450 nm using a Synergy H1 microplate reader (Bio-Tek, Winooski, VT, USA). A target-irrelevant human scFv or IgG_1_, produced in-house using the same procedure as the corresponding test antibody, served as negative control (Supplementary Table S7). All measurements were performed in duplicate, and apparent dissociation constants (K_D_) were determined by nonlinear regression using a one-site binding model in GraphPad Prism.

### Flow cytometry

Flow cytometry was performed to evaluate scFv and IgG binding to membrane-displayed human FPR2 and to assess receptor selectivity across FPR family members. CHO-K1 cells transiently transfected with human FPR2 were harvested 24 h post-transfection, washed three times with PBS, and resuspended in Fluorescence-Activated Cell Sorting (FACS) buffer (PBS containing 0.1% BSA) for antibody staining. For scFv binding assays, 1 × 10^6^ FPR2-overexpressing CHO-K1 cells were incubated with purified scFv at 1 μM for 2 h at room temperature for initial screening. V_L_-engineered scFv variants were tested at 100 nM for 1 h at 4°C using 0.8 × 10^6^ cells. Dose-dependent binding was evaluated using three-fold serial dilutions ranging from 2700 to 3.7 nM. After washing, bound scFvs were detected using iFluor 488–conjugated anti-HA antibody (GenScript, USA) for 1 h at 4°C. For IgG binding assays, FPR2-overexpressing CHO-K1 cells were incubated with purified anti-FPR2 IgG for 1 h at 4°C. Specificity was assessed using CHO-K1 cells transiently expressing FPR1-HA, FPR2-HA, or FPR3-HA constructs, incubated with 10 nM of anti-FPR2 IgG for 1 h at 4°C. Dose–response binding to FPR2-overexpressing CHO-K1 cells and AGS cells was evaluated using 3-fold serial dilutions ranging from 2700 to 0.05 nM. Bound IgGs were detected using Alexa Fluor 488–conjugated anti-human IgG secondary antibody (Thermo Fisher Scientific, USA) for 30 min at 4°C. Finally, cells were washed, resuspended in PBS, and analyzed using a Sony SH800S cell sorter (Sony Biotechnology). Mean fluorescence intensity values were analyzed using FlowJo™ (v10.8.1), and EC_50_ values were determined by nonlinear regression using GraphPad Prism. A target-irrelevant human scFv or IgG_1_, produced in-house using the same procedure as the corresponding test antibody, served as negative controls in all experiments (Supplementary Table S7).

### Western blotting

For the verification of recombinant human FPR family expression, CHO-K1 cells were transiently transfected with pcDNA3.4 vector containing each receptor gene. Cells were harvested 24 h post-transfection and washed twice with PBS (pH 7.4). The cell pellets were lysed in radioimmunoprecipitation assay buffer supplemented with a protease inhibitor cocktail, and supernatants were collected after centrifugation. Total protein concentrations were quantified using the bicinchoninic acid assay.

Equal amounts of total protein (5 μg per lane) were mixed with Dithiothreitol (DTT)-containing sample loading buffer and separated by SDS–PAGE without prior boiling. Proteins were transferred onto nitrocellulose membranes at 100 V for 100 min. After blocking with 5% (w/v) skim milk in 0.05% (v/v) Tris-buffered saline with Tween-20, membranes were incubated overnight at 4°C with either an anti-FPR2 antibody (1:1000), an anti-HA antibody (1:1000), or an anti-β-actin antibody (1:1000). Following washes, membranes were incubated with an HRP-conjugated anti-mouse IgG secondary antibody (1:5000) for 1 h at room temperature and visualized using an enhanced chemiluminescence detection reagent on an imaging system.

### Calcium reporter assay

Intracellular calcium response was monitored using the GCaMP6s fluorescence reporter system [27]. CHO-K1 cells were co-transfected with pGP-CMV-GCaMP6s and pcDNA3.1-FPR2 plasmids and seeded at 8 × 10^4^ cells per well in black, clear-bottom 96-well plates. Cells were incubated overnight at 37°C in 5% CO₂ and washed twice with HBSS–HEPES buffer (Hank’s balanced salt solution supplemented with 25 mM HEPES, pH 7.4) prior to measurement. To evaluate antagonistic activity, cells were pre-incubated with purified anti-FPR2 IgG (0, 10, 100, or 300 nM) or control human IgG_1_ for 45 min at room temperature, washed, and stimulated with 100 nM of Hp(2–20) (GenScript, USA) (Supplementary Table S6). Fluorescence was recorded at excitation 480 nm and emission 510 nm every 5 s for 180 s using a microplate reader. Intracellular calcium responses were quantified as ΔF/F_0_ using Gen5 software and GraphPad Prism.

### Scratch assay

Cell migration was assessed using a scratch wound-healing assay in AGS gastric cancer cells. Cells were seeded at 2.5 × 10^5^ cells/ml in 24-well plates and cultured for 24 h at 37°C in 5% CO₂ to form a confluent monolayer. A linear scratch was introduced using a sterile 200 μl pipette tip, and detached cells were removed by washing with starvation medium (F-12K containing 0.5% FBS). To evaluate inhibitory function, cells were pre-incubated with anti-FPR2 IgG at the indicated concentrations for 1 h prior to stimulation with Hp(2–20) (1 μM) under serum-reduced conditions. A target-irrelevant human IgG_1_ (300 nM) served as negative control (Supplementary Table S7). Images were acquired at 0 and 24 h using a microscope. Wound closure was quantified as the percentage reduction relative to the initial wound area using ImageJ software.

### Transwell migration and invasion assay

Cell migration and invasion were assessed using 24-well Transwell inserts (8 μm pore size; Corning, USA) with AGS gastric cancer cells. For migration assays, 2 × 10^4^ cells suspended in 200 μl starvation medium (F-12K containing 0.5% FBS) were seeded into the upper chamber, and 750 μl starvation medium containing Hp(2–20) (1 μM) was added to the lower chamber. For invasion assays, the upper chamber was pre-coated with Matrigel® Basement Membrane Matrix (growth factor reduced) (200 μg/ml) at 37°C for 2 h prior to cell seeding. Cells were seeded and stimulated under identical conditions as described for migration assays. To evaluate antagonistic activity, cells were pre-incubated with anti-FPR2 IgG at the indicated concentrations for 1 h prior to ligand stimulation. A target-irrelevant human IgG_1_ (300 nM) served as negative control (Supplementary Table S7). After 24 h incubation at 37°C in 5% CO₂, non-migrated or non-invaded cells on the upper membrane surface were removed. Cells on the lower membrane surface were fixed, stained, and quantified by counting five randomly selected fields per insert using ImageJ software.

### Structural modeling

The structure of human FPR2 and anti-FPR2 scFv complex was predicted using AlphaFold 3 server, and the top-ranked model was selected for further analysis based on the interface predicted Template Modeling (ipTM). The selected model was visualized and analyzed using PyMOL software and the key residues and interactions were summarized using LigPlot software.

### Statistical analysis

Statistical analyses were performed using GraphPad Prism 8.0 (GraphPad Software, USA). Comparisons between two groups were analyzed using an unpaired two-tailed Student’s *t-*test, and comparisons among multiple groups were analyzed using one-way Analysis of Variance (ANOVA) followed by Tukey’s post hoc test. All quantitative data are presented as the mean ± SD, and differences were considered statistically significant at *P* < .05.

## Results

### Construction of a CDR-H3-focused synthetic human scFv library

We constructed a fully human scFv library based on the human germline pair VH3-23 and Vκ1-39, which were selected due to their prevalence in human repertoires and favorable biophysical properties. These scaffolds have been reported to support robust expression in both bacterial and mammalian systems, show high phage-display efficiency, and maintain substantial thermal stability, providing a suitable backbone for iterative selective campaigns [28, 29].

To design a CDR-H3 library optimized for FPR2 ECL3 engagement, sequence diversity was preferentially introduced into the side regions of the CDR-H3 loop to favor interactions with the ECL3 peptide. The degenerate codons were selected to encode amino acids with physicochemical properties compatible with the FPR2 ECL3 region, while also considering the negatively charged environment and structural features of the FPR2 ligand-binding pocket. This rational CDR-H3 design generated a theoretical diversity of 9.67 × 10^8^ variants (Supplementary Table S1). The library was assembled through overlap-extension PCR using degenerated primers, followed by an in-frame selection step based on β-lactamase fusion to eliminate sequences containing frameshifts or premature stop codons [25]. This process resulted in 1.30 × 10^9^ CFU with a 100% in-frame rate among randomly sequenced clones, exceeding the theoretical diversity and indicating comprehensive coverage of the designed CDR-H3 variants.

After subcloning into a phagemid vector and helper-phage rescue, the final phage-displayed scFv library achieved a titer of 1.25 × 10^15^ CFU while maintaining complete in-frame integrity (Supplementary Fig. S1 and Supplementary  Table  S2). These results confirm the successful generation of a high-quality synthetic CDR-H3-focused human scFv library suitable for downstream antibody selection.

### Isolation of anti-FPR2 antibodies binding to extracellular loop 3

**Figure 1:**
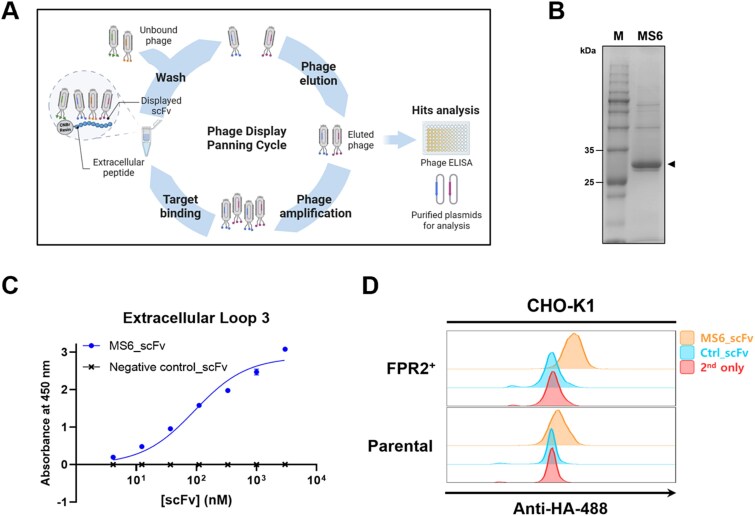
Generation of anti-FPR2 antibody. (A) Schematic illustration of phage display biopanning cycles using FPR2 ECL-mimicking peptides as antigen to screen FPR2 antibodies. (B) SDS-PAGE analysis of the isolated anti-FPR2 scFv (MS6) after purification. (C) Analysis of the binding activity of MS6 scFv to human FPR2 ECL3 peptide using ELISA. (D) Flow cytometric analysis of MS6 scFv binding on FPR2 overexpressing CHO-K1 cells. A target-irrelevant human scFv served as control. Error bars represent mean ± SD from technical replicates (*n* = 2).

To identify antibodies recognizing FPR2 ECL3, we performed solution-phase phage-display biopanning using a synthetic ECL3 peptide conjugated to CNBr-activated resin (Fig. 1A). Five rounds of panning were performed under increasing selection stringency, and the detailed panning conditions, phage titers, and enrichment folds are summarized in Supplementary Table S3. A unique scFv clone with the most favorable binding profile was isolated and termed MS6. MS6 scFv was subsequently expressed in bacteria, purified, and subjected to functional characterization (Fig. 1B).

ELISA confirmed dose-dependent binding to ECL3 peptide, yielding an apparent K_D_ of 92.9 nM (Fig. 1C). Flow cytometric analysis further showed that MS6 scFv staining produced a greater fluorescence peak shift in FPR2-overexpressing CHO-K1 cells than in parental CHO-K1 cells, confirming recognition of the membrane embedded receptor by MS6 scFv (Fig. 1D). FPR2 expression in transiently transfected CHO-K1 cells was confirmed using a commercial anti-FPR2 antibody (Supplementary Fig. S2). These data indicate that the epitope identified from the peptide is exposed and structurally preserved on the full-length receptor, supporting the suitability of this approach for isolating physiologically relevant binders.

### Engineering of the V_L_ domain of anti-FPR2 antibodies for extracellular loop 2 engagement

**Figure 2:**
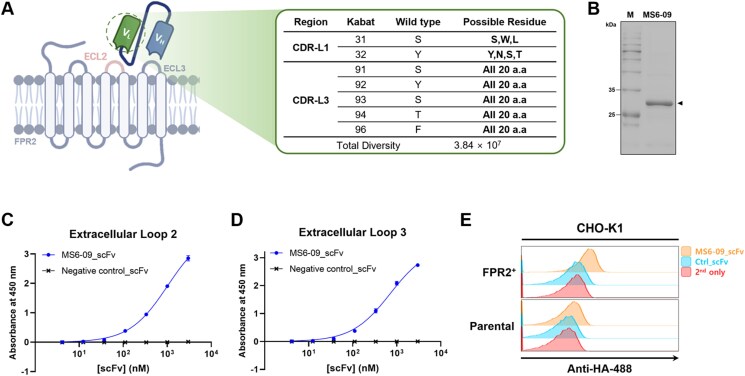
Identification of VL variants with dual-ECL binding properties. (A) Schematic representation of the antibody engineering strategy using site-directed and saturation mutagenesis. Residues are represented by one-letter amino acid codes. a.a, amino acids. (B) SDS-PAGE analysis of the engineered anti-FPR2 scFv (MS6-09) after purification. (C and D) Analysis of the binding profiles of MS6-09 scFv to human FPR2 ECL2 peptide (C) and ECL3 peptide (D) using ELISA. (E) Flow cytometric analysis of MS6-09 scFv binding to FPR2 overexpressing CHO-K1 cells. A target-irrelevant human scFv served as control. Error bars represent mean ± SD from technical duplicates.

Given that ECL2 and ECL3 collectively frame the extracellular entrance of the orthosteric ligand-binding pocket of FPR2 [30, 31], engagement of both loops was hypothesized to sterically hinder ligand access and enhance antagonistic potential. Accordingly, we next engineered the light-chain variable region of MS6 to enable additional recognition of FPR2 ECL2, while preserving the V_H_ domain. Site-directed mutagenesis was applied to CDR-L1, and saturation mutagenesis was introduced across CDR-L3, while conserving structurally stabilizing residues (Fig. 2A) [32]. This focused diversification strategy was designed to introduce complementary contacts with ECL2 without disrupting the structural integrity of the parental framework. The designed library yielded a theoretical diversity of 3.84 × 10^7^ unique V_L_ variants. The resulting V_L_-diversified scFv library was constructed and subsequently displayed on phage as described above (Supplementary Fig. S1), achieving a final titer of 1.74 × 10^15^ CFU with complete in-frame integrity (Supplementary Table S4).

### Isolation of dual-loop engaging anti-FPR2 antibodies

To isolate V_L_ variants capable of ECL2 engagement, the constructed V_L_-diversified scFv library was screened using a synthetic peptide corresponding to the human FPR2 ECL2 region as antigen. Three rounds of panning were conducted under increased selection stringency, with detailed panning conditions, phage titers, and enrichment folds summarized in Supplementary Table S5. Following clone screening, a unique V_L_ variant of MS6, MS6-09, exhibiting the most favorable binding properties, was selected for further characterization.

The selected clone was expressed and purified in a bacterial system (Fig. 2B) and evaluated for binding to both ECL2 and ECL3 peptides by ELISA. MS6-09 scFv demonstrated dose-dependent binding to both peptides with apparent K_D_ of 1016 nM for ECL2 (Fig. 2C) and 710.9 nM for ECL3 peptide (Fig. 2D). As a result, ECL3 binding was maintained following V_L_ diversification, while additional recognition of ECL2 was conferred, consistent with successful epitope expansion without loss of the primary binding interface.

To determine whether peptide reactivity translated into recognition of the native receptor, binding to membrane-expressed FPR2 was evaluated by flow cytometry in FPR2-overexpressing CHO-K1 cells. A fluorescence shift was detected in FPR2-overexpressing CHO-K1 cells compared with both the control antibody and parental CHO-K1 cells, demonstrating specific recognition of the native receptor conformation (Fig. 2E). Together, these findings establish the generation of a dual-loop engaging antibody capable of recognizing FPR2 in its membrane context.

### IgG reformatting and binding properties of the dual-loop engaging anti-FPR2 antibody

**Figure 3:**
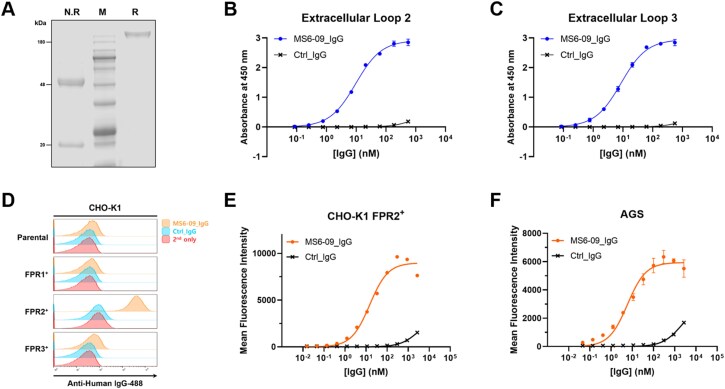
Characterization of anti-FPR2 IgG. (A) SDS-PAGE analysis of purified anti-FPR2 IgG (MS6-09) under non-reducing (N.R) and reducing (R) conditions. M, molecular weight marker. (B and C) Analysis of the dual-loop binding profiles of MS6-09 IgG to human FPR2 ECL2 (B) and ECL3 peptide (C) using ELISA. Values are presented as mean ± SD from technical duplicates. (D) FACS histograms showing binding specificity of MS6-09 IgG toward FPR family receptors. (E and F) Flow cytometric analysis showing dose–response binding curves of MS6-09 IgG on FPR2-overexpressing CHO-K1 cells (E) and FPR2-positive AGS gastric cancer cells (F) for apparent binding EC50 determination. A target-irrelevant human IgG1 served as a control. Values are presented as mean ± SD from two independent experiments.

To evaluate the therapeutic efficacy, MS6-09 was reformatted into a human IgG_1_ using a mammalian expression system (Fig. 3A). Binding of anti-FPR2 IgG to FPR2 ECL2 and ECL3 peptides was next assessed by ELISA. Dose-dependent binding was observed for both peptides, consistent with retention of dual-loop recognition after IgG reformatting. Nonlinear regression analysis of the binding curves yielded an apparent K_D_ of 9.6 nM for ECL2 (Fig. 3B) and 8.6 nM for ECL3 (Fig. 3C).

Given the high sequence homology among FPR family members, selectivity of engineered IgG was evaluated across FPR1, FPR2, and FPR3 using CHO-K1 cells transiently expressing each receptor using flow cytometry. Expression of each FPR family receptor in transiently transfected CHO-K1 cells was validated by western blotting (Supplementary Fig. S3). The engineered IgG exhibited selective binding to FPR2-expressing cells, whereas no detectable fluorescence signals were observed in FPR1- or FPR3-expressing cells as well as in parental cells (Fig. 3D). In addition, binding curve analysis demonstrated dose-dependent binding of the engineered IgG to both FPR2-expressing CHO-K1 cells and endogenous FPR2-positive AGS gastric cancer cells (Supplementary Fig. S4), with EC_50_ values of ~15 (Fig. 3E) and 5.9 nM (Fig. 3F), respectively. Taken together, these results indicate that the antibody effectively targets FPR2-expressing GC cells, supporting its potential application in GC progression.

### Antagonistic activity of anti-FPR2 antibody on FPR2-mediated signaling and gastric cancer progression

**Figure 4:**
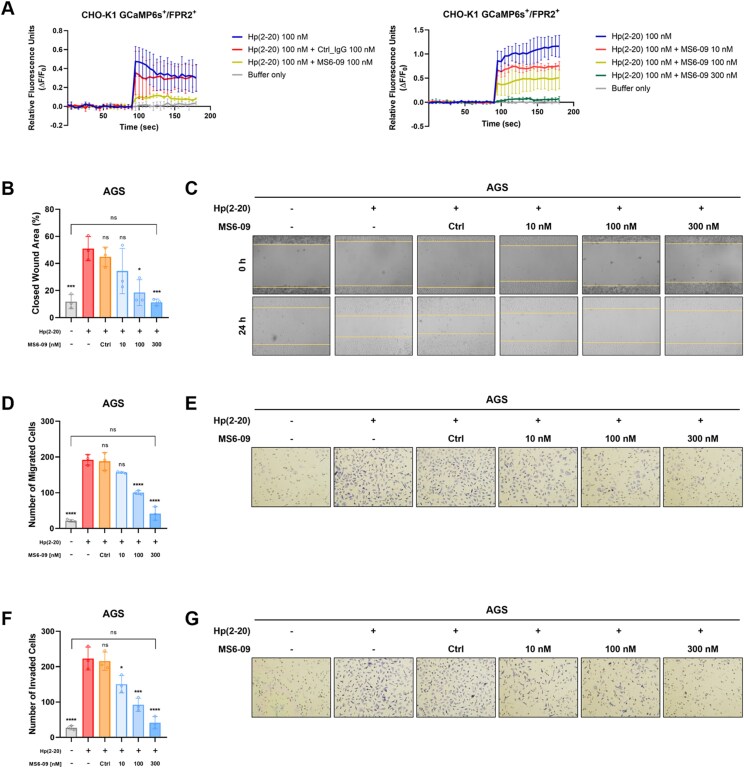
Antagonistic activity of anti-FPR2 IgG. (A) Inhibitory function of anti-FPR2 IgG MS6-09 on ligand induced calcium response in CHO-K1 cells co-expressing FPR2 and GCaMP6s. Cells were pre-incubated with MS6-09 IgG at the indicated concentrations prior to stimulation with Hp(2–20) at a concentration of 100 nM. (B and C) Inhibitory function of MS6-09 IgG on ligand-induced wound recovery of AGS cells. Quantification of wound closure at 24 h relative to the initial wound area in % (B), and the representative images of wound closure (marked by yellow lines) on confluent monolayers of AGS cells at 0 and 24 h (C). (D and E) Inhibitory function of MS6-09 IgG on ligand-induced migration of AGS cells. Quantification of migrated cells (D) and the representative images of migrated cells after 24 h (E). (F and G) Inhibitory function of MS6-09 IgG on ligand-induced invasion of AGS cells. Quantification of invaded cells (F) and the representative images of invaded cells after 24 h (G). Cells were stimulated with Hp(2–20) at a concentration of 1 μM unless otherwise indicated. A target-irrelevant human IgG1 was used as a control at 100 nM in panel A and at 300 nM in panels B–G. All data are presented as the mean ± SD from three independent experiments. ns (not significant), ^*^P < .05; ^**^P < .01; ^***^P < .001; ^****^P < .0001.

Beyond receptor targeting, the inhibitory function of MS6-09 IgG on FPR2 activation was next validated. First, FPR2 signaling was assessed using a GCaMP6s-based calcium reporter assay [27] in CHO-K1 cells co-expressing human FPR2. Stimulation with Hp(2–20) induced a rapid increase in intracellular calcium levels, whereas pre-incubation with MS6-09 IgG attenuated the ligand-triggered response relative to control antibody, with a dose-dependent reduction in fluorescence observed across increasing antibody concentrations (Fig. 4A). These results indicate that the MS6-09 IgG effectively antagonizes Hp(2–20)–mediated FPR2 activation at the signaling level.

As FPR2 activation promotes pro-migratory responses in GC, the effect of the MS6-09 IgG on GC cell migration was subsequently examined. Cell motility was first assessed using a scratch assay in AGS cells. Treatment with Hp(2–20) markedly accelerated wound closure over 24 h compared with unstimulated controls, indicating increased cell motility. Pre-incubation with the MS6-09 IgG prior to ligand stimulation attenuated Hp(2–20)–induced wound closure in a dose-dependent manner, whereas control antibody did not significantly affect cell motility (Fig. 4B and C). Quantitative analysis of the wound closure confirmed suppression of Hp(2–20)–driven cellular motility following antibody treatment.

To further evaluate migration and invasion, transwell assays were performed under identical stimulation conditions. Treatment with Hp(2–20) increased the number of AGS cells migrating through the membrane compared with unstimulated controls. Pre-incubation with MS6-09 IgG reduced Hp(2–20)–induced migration in a dose-dependent manner, whereas control antibody did not significantly alter the migratory response (Fig. 4D and E). Similarly, Hp(2–20) treatment enhanced invasion of AGS cells, and this increase was reduced in a dose-dependent manner following pre-incubation with MS6-09 IgG, while control antibody showed no measurable effect (Fig. 4F and G). Quantification of migrated and invaded cells confirmed attenuation of ligand-induced migration and invasion following antibody treatment, consistent with the effects observed in the scratch assay. Collectively, these results indicate antagonistic function of MS6-09 IgG toward FPR2 in GC cells.

### Structure-guided insights into the antagonistic mechanism of the anti-FPR2 antibody

**Figure 5:**
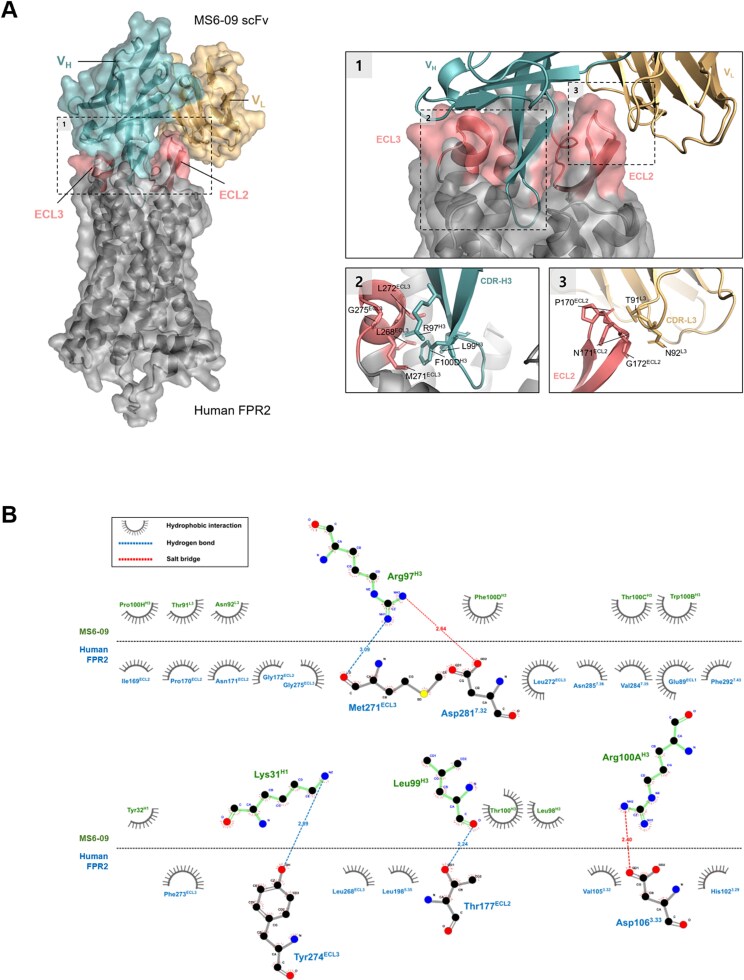
Structural insights into the antagonistic activity of anti-FPR2 antibody. (A) Predicted docking model of FPR2-MS6-09 scFv complex. Zoomed-in views of regions 1–3 show putative interactions between MS6-09 and the ECLs of FPR2. Region 1 shows the predicted extracellular binding interface, while regions 2 and 3 highlight key CDR-mediated interactions with ECL3 and ECL2, respectively. (B) Comprehensive interaction profile visualized as a LigPlot interaction diagram.

To elucidate the molecular basis of epitope recognition and antagonistic activity of MS6-09, *in silico* docking analysis was performed. A complex model of human FPR2 and the anti-FPR2 scFv was generated using AlphaFold 3 [33], and the top-ranked prediction (pTM = 0.59; ipTM = 0.3) was selected for structural analysis. The predicted interface was visualized using PyMOL, and potential residue-level interactions were summarized using LigPlot (Fig. 5A) [34].

The docking model suggests that the antibody engages FPR2 primarily through interactions with ECL3 and ECL2. CDR-H3 appeared to constitute a major interaction interface with ECL3 residues. Within this interface, Arg97^CDR-H3^ was positioned near Met271^ECL3^, suggesting a possible polar contact, while multiple CDR-H3 residues were predicted to contribute to hydrophobic interactions with Leu268^ECL3^, Leu272^ECL3^, and Gly275^ECL3^. The V_L_ domain contributed to complementary interactions with ECL2. Residues Thr91^CDR-L3^ and Asn92^CDR-L3^ were positioned near Pro170^ECL2^, Asn171^ECL2^, and Gly172^ECL2^, forming a putative hydrophobic interaction cluster consistent with the aim of the engineering strategy. Additional contacts, including possible salt bridges and multiple polar interactions, were observed within the upper transmembrane (TM) regions proximal to the extracellular entrance (Fig. 5B).

Collectively, this binding configuration positions the antibody across the extracellular entrance of the orthosteric ligand-binding pocket, providing a structural rationale for steric blockade of Hp(2–20) binding and inhibition of FPR2 activation.

## Discussion

FPR2 participates in diverse inflammatory and tumor-promoting pathways, and increasing evidence implicates dysregulated activation of the Hp(2–20)–FPR2 axis in promoting migratory and invasive phenotypes in gastric cancer cells [7, 11]. Despite the therapeutic potential of targeting this receptor, antibody-based antagonists remain limited. Therefore, we sought to develop a fully human antagonistic antibody capable of functionally inhibiting FPR2 signaling within a gastric cancer context.

Through a stepwise engineering strategy, we established a dual-loop engaging FPR2 antibody with functional antagonistic activity. Initial biopanning against an ECL3-mimicking peptide yielded a scFv clone that bound the peptide in a dose-dependent manner and recognized the native receptor on transfected CHO-K1 cells. To enhance antagonistic efficacy, we next engineered the V_L_ domain to extend epitope recognition to ECL2 while maintaining ECL3 binding. Because ECL2 and ECL3 together form the extracellular vestibule of FPR2 orthosteric pocket, concomitant engagement of both loops was anticipated to impose steric hindrance on ligand access.

V_L_-domain diversification successfully introduced ECL2 reactivity while preserving ECL3 binding. Although the engineered scFv variants exhibited lower apparent affinity than the parental clone—an expected trade-off during epitope expansion rather than affinity optimization—the ability to bind membrane-displayed FPR2 was retained, indicating that V_L_ diversification did not compromise functional receptor engagement.

Conversion of the engineered scFv into a human IgG_1_ format maintained recognition of both ECL2 and ECL3. The lower apparent K_D_ values observed in the IgG format may reflect not only avidity enhancement through bivalent binding, but also improved structural stability and functional presentation in the IgG format. Apparent affinity values vary across assays, as expected, but consistent binding to peptides, receptor-overexpressing cells, and endogenous FPR2-positive AGS cells confirmed that the IgG maintained its engineering specificity. Notably, despite high homology among FPR family members [35], the antibody exhibited selective binding to FPR2 over FPR1 and FPR3, underscoring its target specificity.

Given the structural similarity among FPR family members, selection based on a single ECL peptide may carry an inherent risk of enriching clones that recognize conserved determinants shared across related receptors. In this context, incorporation of an additional ECL as an antigenic determinant was intended to increase epitope complexity and reduce the likelihood of cross-reactivity. While the present study did not directly compare selectivity profiles between single-loop and dual-loop binders, the observed FPR2-selective binding is compatible with this design rationale. Functionally, the antibody attenuated Hp(2–20)-induced intracellular calcium mobilization in a GCaMP6s reporter system and suppressed Hp(2–20)-driven wound closure, migration, and invasion in AGS gastric cancer cells. These *in vitro* findings indicate that the antibody possesses inhibitory activity and support further *in vivo* evaluation of this antibody for therapeutic application. Accordingly, further downstream signaling analyses, including MAPK/ERK and AKT phosphorylation, together with additional functional characterization using FPR1- or FPR3-expressing cells, are currently planned as follow-up investigations to more comprehensively define the pharmacological profile, receptor selectivity, and molecular mechanism underlying the antagonistic activity of MS6-09. *In vivo* studies in relevant disease models are also planned to evaluate its therapeutic potential.

A predicted MS6-09–FPR2 complex model suggested a possible binding arrangement consistent with the dual-loop engagement approach, involving CDR-H3–ECL3 contacts complemented by V_L_-mediated interactions with ECL2. However, the predicted complex showed a relatively low ipTM score, which may reflect a fundamental modeling limitation arising from the intrinsic flexibility of GPCR ECLs and antibody CDR loops. Therefore, this model should be interpreted as a hypothetical structural model.

Although peptide-based antigen strategies are frequently employed in GPCR antibody discovery, they cannot be universally generalized to all GPCR targets. Extracellular loop architecture varies considerably among GPCR family members, with differences in loop length, glycosylation, disulfide bonding patterns, and conformational coupling to transmembrane domains [23, 36]. Thus, the success of the present approach likely reflects structural features specific to FPR2 and the accessibility of its ECLs, rather than a universally applicable GPCR-targeting platform. Careful structural consideration remains essential when extending similar strategies to other GPCR targets.

Overall, this work demonstrates a rational engineering strategy that enables the development of a fully human dual-loop-targeting antibody with potent antagonistic activity toward FPR2, offering for future therapeutic exploration in gastric cancer and other FPR2-related pathologies.

## Supplementary Material

Revised_Supplementary_material_tbag035

## Data Availability

All data supporting the findings of this study are available within the article and Supplemental Information. Raw data files are available by reasonable request to the corresponding author.
