# Supplementary material for: Fully human antagonistic antibodies targeting FPR2 through dual extracellular-loop engagement for gastric cancer therapy
Source: Antib Ther. 2026 Jul 3;9(3):449–59. doi: 10.1093/abt/tbag035 (PMC13463766; doi:10.1093/abt/tbag035)
Supplement: Supplementary file 1 [file revised_supplementary_material_tbag035.docx]

**Fully Human Antagonistic Antibodies Targeting FPR2 Through Dual Extracellular-Loop Engagement for Gastric Cancer Therapy**

Min Su Kim^1,2^, Tae Hyun Kang^1,2,3,4,*^

^1^ Department of Biopharmaceutical Chemistry, Kookmin University, Seoul, Republic of Korea

^2^ Antibody Research Institute, Kookmin University, Seoul, Republic of Korea

^3^ Biopharmaceutical Chemistry Major School of Applied Chemistry, Kookmin University, Seoul, Republic of Korea

^4^ Department of Pharmaceutical Engineering, Kookmin University, Seoul, Republic of Korea

^*^ Correspondence: [thkang@kookmin.ac.kr](mailto:thkang@kookmin.ac.kr)

**Figure S1. Schematic overview of the construction of the CDR-H3-focused scFv library**

**Figure S2. Flow cytometry analysis of human FPR2 expression on transiently transfected CHO-K1 cells.** CHO-K1 cells transiently transfected with human FPR2 were stained with a commercial anti-FPR2 primary antibody followed by a fluorescently labeled secondary antibody (solid line). The secondary antibody–only control is shown as a dashed line.

**Figure S3. Validation of human FPR family expression levels on transiently transfected CHO-K1 cells.** Western blot analysis of C-terminal HA tagged FPR1 (A), FPR2 (B), and FPR3 (C) expression in transiently transfected CHO-K1 cells.

**Figure S4. Flow cytometry analysis of human FPR2 expression on AGS cells.** AGS cells were stained with a commercial anti-FPR2 primary antibody followed by a fluorescently labeled secondary antibody (solid line). The secondary antibody-only control is shown as a dashed line.

**Table S1. Design of CDR-H3 library**

| **Kabat** | **Codon^*^** | **Possible Residue^**^** |
| --- | --- | --- |
| 93 | GCA | A |
| 94 | AAA | K |
| **95** | **AVC** | **N/S/T** |
| **96** | **DHC** | **A/D/F/I/N/S/T/V/Y** |
| **97** | **VRK** | **D/E/G/H/K/N/Q/R/S** |
| **98** | **NTT** | **F/I/L/V** |
| **99** | **KBG** | **A/G/L/S/V/W** |
| **100** | **AVC** | **N/S/T** |
| **100A** | **ARA** | **R/K** |
| **100B** | **KKG** | **G/L/V/W** |
| **100C** | **AVG** | **R/K/T** |
| **100D** | **NTT** | **F/I/L/V** |
| **100E** | **DHC** | **A/D/F/I/N/S/T/V/Y** |
| **100F** | **KKG** | **G/L/V/W** |
| **100G** | **AST** | **S/T** |
| **100H** | **NYT** | **A/F/I/L/P/S/T/V** |
| 100I | TTC | F |
| 101 | GAC | D |
| 102 | TAC | Y |
| **Total Diversity** | | **9.67 × 10^8^** |

* Codons represent nucleotide or degenerate codons used for mutagenesis.

** Possible residues are represented by one-letter amino acid codes.

Positions diversified within the CDR region are indicated in bold.

**Table S2. Summary of the constructed CDR-H3-focused scFv libraries.**

Library size was determined by colony-forming unit (CFU) counting, and in-frame rates were evaluated by sequencing randomly selected colonies.

| **Library** | **Size (CFU)** | **In-frame rate** |
| --- | --- | --- |
| Designed scFv Library | 9.67 × 10^8^ | - |
| pBLA scFv Library | 1.30 × 10^9^ | 100% (20/20) |
| pComb3X scFv Library | 1.10 × 10^10^ | 100% (19/19) |
| Phage-scFv Library | 1.25 × 10^15^ | 100% (18/18) |

**Table S3. Summary of bio-panning rounds for FPR2 ECL3**

| **Round** | **Antigen (μg)** | **Wash Condition** | **Input Phage**  **(CFU/mL)** | **Output Phage**  **(CFU/mL)** | **Enrichment**  **(Fold)** |
| --- | --- | --- | --- | --- | --- |
| 1 | 10 | 1X PBS × 3 | 1.25 × 10^15^ | 3.83 × 10^8^ | 1 |
| 2 | 10 | 0.05% PBST × 3 | 6.50 × 10^15^ | 9.29 × 10^9^ | 4.67 |
| 3 | 10 | 0.05% PBST × 5 | 9.29 × 10^9^ | 1.67 × 10^6^ | 585 |
| 4 | 5 | 0.1% PBST × 5 | 2.68 × 10^13^ | 2.00 × 10^10^ | 2433 |
| 5 | 5 | 0.1% PBST × 7 | 1.93 × 10^16^ | 4.17 × 10^9^ | 0.704 |

**Table S4. Summary of the constructed V_L_-diversified scFv libraries.**

Library size was determined by colony-forming unit (CFU) counting, and in-frame rates were evaluated by sequencing randomly selected colonies.

| **Library** | **Size (CFU)** | **In-frame rate** |
| --- | --- | --- |
| Designed scFv Library | 3.84 × 10^7^ | - |
| pBLA scFv Library | 7.70 × 10^8^ | 100% (20/20) |
| pComb3X scFv Library | 9.80 × 10^8^ | 100% (17/17) |
| Phage-scFv Library | 1.74 × 10^15^ | 100% (21/21) |

**Table S5. Summary of bio-panning rounds for FPR2 ECL2**

| **Round** | **Antigen (μg)** | **Wash Condition** | **Input Phage**  **(CFU/mL)** | **Output Phage**  **(CFU/mL)** | **Enrichment**  **(Fold)** |
| --- | --- | --- | --- | --- | --- |
| 1 | 20 | 1X PBS × 3 | 1.74 × 10^15^ | 3.83 × 10^8^ | 1.0 |
| 2 | 20 | 0.05% PBST × 3 | 3.25 × 10^14^ | 3.96 × 10^8^ | 5.5 |
| 3 | 20 | 0.05% PBST × 5 | 3.96 × 10^8^ | 1.25 × 10^5^ | 1433 |

**Table S6. Amino acid sequences of peptides used in this study**

| **Peptide** | **Amino acid sequence** |
| --- | --- |
| FPR2 ECL2 | TTVTIPNGDTYCTFNFASWGG |
| FPR2 ECL3 | LKEMLFYGK |
| Hp(2-20) | AKKVFKRLEKLFSKIQNDK |

**Table S7. CDR amino acid sequences of antibodies used in this study**

| **Antibody** | **CDR Amino acid sequence (IMGT)** | | | | | |
| --- | --- | --- | --- | --- | --- | --- |
|  | H1 | H2 | H3 | L1 | L2 | L3 |
| MS6 | GFTFSKYA | ISGSGGST | AKTSRLLTRWTFAVSPFDY | QSISSY | AAS | QQSYSTP |
| MS6-09 | - | - | - | QSISSN | - | QQTNETP |
| Negative control | GFTFSDHA | ISYNGSNT | AKGLTTANFFPFDY | SSNIGSNA | DNN | GSWDDSLNAYV |
